# Probable Association Between Hidradenitis Suppurativa and Inflammatory Bowel Disease: A Case Report

**DOI:** 10.7759/cureus.111488

**Published:** 2026-06-25

**Authors:** Mason Arbabi, Hafiz Muhammad Faizan Mughal, Atiq Ul Rehman, Abdul Hanan, Saviz Saghari

**Affiliations:** 1 Internal Medicine, University of Kentucky, Lexington, USA; 2 Internal Medicine, Khawaja Muhammad Safdar Medical College, Sialkot, PAK; 3 Medicine, Khawaja Muhammad Safdar Medical College, Sialkot, PAK; 4 Internal Medicine, West Anaheim Medical Center, Anaheim, USA

**Keywords:** diverticulitis, hidradenitis suppurativa, inflammatory bowel disease, proctitis, rectal bleeding

## Abstract

Hidradenitis suppurativa (HS) is a chronic, inflammatory skin disease characterized by recurrent painful nodules, abscesses, and sinus tracts primarily affecting intertriginous areas, causing significant quality of life impairment. Follicular occlusion, microbial infection, aberrant immune responses, and genetic and environmental factors are all recognized as playing a significant role in its complex pathogenesis. On the other hand, inflammatory bowel disease (IBD) is a relapsing and remitting disorder characterized by chronic inflammation at various sites in the gastrointestinal tract. The pathogenesis of IBD is a multifaceted interplay between genetic susceptibility, dysregulated immune responses, environmental triggers, and gut microbiota alterations, or dysbiosis. Herein, we present a case of a 64-year-old man with longstanding HS, chronic colostomy, and multiple comorbidities who presented with acute bright red blood per rectum. Our extensive workup revealed multiple perianal open fistulous tracts with scarring, large external hemorrhoids, active chronic proctitis with focal crypt abscess formation and erosion, and diverticulitis with negative infectious workup. His HS remained active throughout the hospitalization. Given the combination of chronic skin lesions, active proctitis on histology, and elevated inflammatory markers, these findings raised concern for underlying IBD, although definitive diagnosis required further outpatient evaluation. This report explores the diagnostic challenges at the crossroads of HS and potential IBD.

## Introduction

Hidradenitis suppurativa (HS) is a chronic, disabling skin disorder, characterized by deep tender subcutaneous nodules that lead to fibrosis and extensive sinus tracts mainly affecting areas with apocrine glands [1]. The point prevalence of HS in the United States of America (USA) is 0.5-4% (variable in different studies), while the one-year prevalence is around 1% [2]. By the 1990s, hair follicle blockage was widely accepted as the primary cause of HS [3]. Several other factors, including genetics, biofilms, bacterial infection, hormone imbalance, age, gender, smoking, obesity, lifestyle factors, and medications, have been studied for their role in the development and progression of HS. A 2023 genome-wide association study (GWAS) found genetic variants near the SOX9 (SRY-Box transcription factor 9) and KLF5 (Krüppel-like factor 5) genes associated with HS [4]. Evidence strongly suggests a central role for immune dysregulation in HS, involving an abnormal activation of both the innate and adaptive immune systems [5].

Inflammatory bowel disease (IBD) refers to chronic, relapsing inflammatory conditions of the digestive tract, with the two main types being Crohn’s disease (CD) and ulcerative colitis (UC). Several risk factors, including genetics, smoking, obesity, diet, air pollution, stress, certain medications, age, race, and ethnicity, can contribute to the development of IBD [6]. Strong evidence indicates that in genetically susceptible individuals, an aberrant immune response to an altered gut microbiota (dysbiosis) is a key driver of IBD [7]. In a large meta-analysis, smoking is found to be a significant risk factor for CD. As we mentioned, obesity, particularly severe obesity, is increasingly recognized as a risk factor for developing IBD; however, the high prevalence of obesity in patients with IBD might also suggest an independent effect of IBD on the risk of obesity development [8].

IBD, especially CD, is a frequently reported comorbidity in patients with HS [9]. HS shares common clinical manifestations, genetic susceptibility, and immunological features with IBD [10]. Both HS and IBD can cause painful abscesses, sinus tracts, and scarring, which can make it difficult to distinguish between the two diseases when they occur in the same area. Both diseases have strong hereditary components, with many patients reporting a family history. Genetic evaluations have identified several specific genes involved in both diseases. Sulfotransferase family 1B member 1 (SULT1B1) and sulfotransferase family 1E member 1 (SULT1E1) genes encode sulfotransferase enzymes that help regulate the body's response to hormones and other compounds [11]. The GWAS study has identified variants in or near the SULT1B1 and SULT1E1 genes as potential risk factors for HS in the context of IBD [12]. The elongation of very long-chain fatty acids-like 7 (ELOVL7) gene has also been identified as having a possible protective association against HS in individuals who also have IBD [13]. HS and IBD are driven by similar overactive inflammatory pathways, involving the same key immune system molecules. Both diseases are characterized by elevated levels of proinflammatory cytokines, including tumor necrosis factor-alpha (TNF-α), interleukin-1 beta (IL-1β), IL-12, IL-17, and IL-23 [14,15]. An emerging literature review reinforces shared cytokine profiles between HS and IBD, particularly implicating the IL-23 and T-helper 17 (Th17) axis [15].

This case highlights the diagnostic and therapeutic crossroads faced when HS, proctitis, diverticulitis, and potential IBD intersect in a patient with a remote colostomy and multiple comorbidities.

## Case presentation

This case highlights a 64-year-old Caucasian man with a history of class III obesity (Body Mass Index of 41), type 2 diabetes mellitus, hypertension, dyslipidemia, chronic obstructive pulmonary disease on 2-3 L oxygen via nasal cannula, and longstanding HS affecting the axillary, inguinal, abdominal wall, and sacral regions (Figure 1).

**Figure 1:**
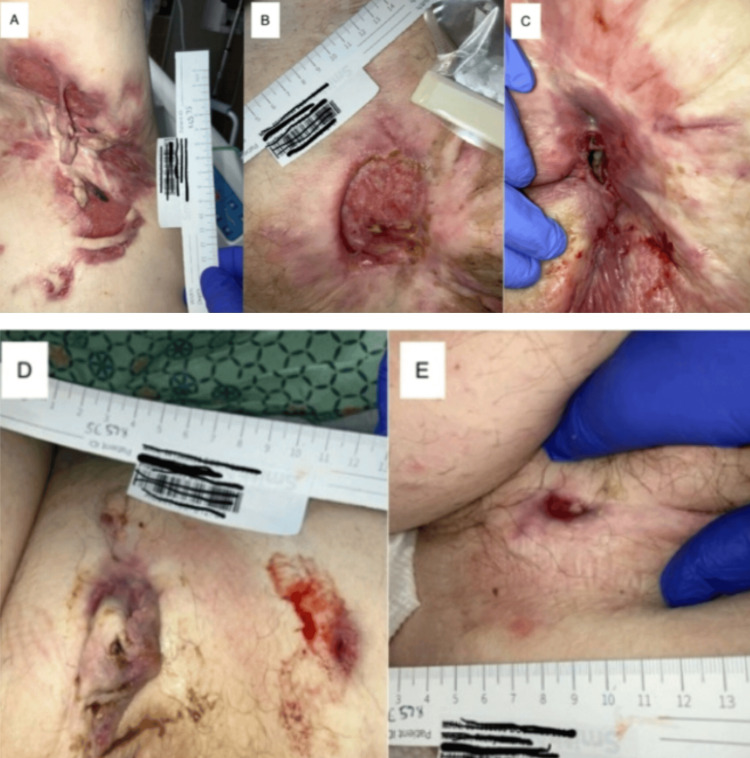
The patient was suffering from chronic hidradenitis suppurativa affecting the axillary (Panel A), inguinal (Panel E, right groin; Panel D, left groin), abdominal wall (Panel B, right lower abdominal wall), and sacral (Panel C) areas. The "long standing" nature of the condition indicates a chronic course where hair follicles become blocked, leading to inflammation, painful abscesses, and the formation of tunnels under the skin (sinus tracts). These can then develop into fistulas, which are persistent tunnels that drain pus or blood. The scarring is a result of the chronic inflammation and repeated lesions.

He presented to the emergency department (ED) with bright red bleeding per rectum. The patient had undergone a colostomy in 1995 for a presumed colonic mass and perianal HS; the original pathology report was unavailable. He reports experiencing a sudden onset of bright red blood per rectum without abdominal pain, nausea, vomiting, or changes in bowel habits. In the ED, he was tachycardic and had leukocytosis on laboratory testing but was afebrile on exam. His physical examination revealed an active flare of HS with multiple draining sinuses in the armpits, groin, and sacrum; his stoma appeared healthy, and the abdomen was nontender. Initial vital signs on admission revealed a temperature of 37.1 °C, a blood pressure of 138/82 mmHg, a pulse rate of 110/minute, and a respiratory rate of 18/minute with an oxygen saturation of 99% on 2 L oxygen via nasal canula. A contrast-enhanced abdominal/pelvic computed tomography (CT) scan from an outside facility demonstrated chronic sigmoid diverticulitis and a 4.5×3.0×3.5 cm nodular density in the proximal sigmoid colon, raising concerns for malignancy versus chronic inflammation. A repeat abdominal/pelvic CT scan confirmed these findings (Figure 2) and gastroenterology was consulted. 

**Figure 2:**
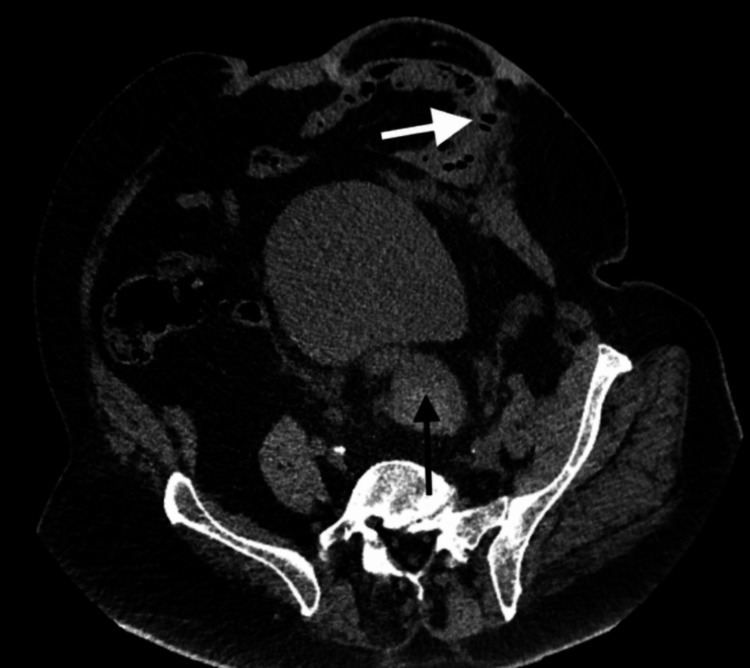
Contrast-enhanced abdominal/pelvic computed tomography (CT) scan showed chronic sigmoid diverticulitis (White arrow) and a 4.5×3.0×3.5 cm nodular density in the proximal sigmoid colon (Black arrow), raising concerns for malignancy versus chronic inflammation.

Flexible sigmoidoscopy was performed and extensive perianal scarring with severe open fistulous tracts, large external hemorrhoids, severely erythematous, friable mucosa with erosions, loss of vascular pattern, and pseudo-membranes in the rectum and rectosigmoid colon were identified. Additionally, extensive diverticulosis was present in the splenic flexure, descending colon, sigmoid colon, and rectosigmoid colon, and three bleeding diverticula were detected in the rectosigmoid colon, which were successfully clipped (Figure 3).

**Figure 3:**
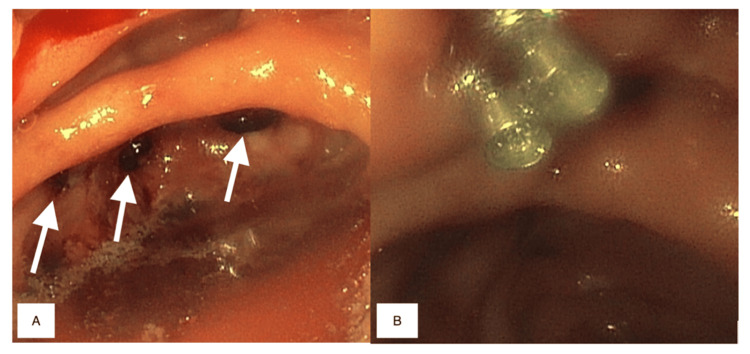
Flexible sigmoidoscopy revealed extensive diverticulosis in the splenic flexure, descending colon, sigmoid colon, and rectosigmoid colon. Three bleeding diverticula (White arrows) were identified in the rectosigmoid colon (Panel A), which were clipped successfully (Panel B).

Biopsies obtained during sigmoidoscopy showed moderately active chronic proctitis with focal crypt abscess formation and mucosal erosion. Findings were negative for dysplasia or granulomas, as well as special stains for bacteria, fungi, and acid-fast organisms. Inflammatory markers revealed a markedly elevated fecal calprotectin and an elevated fecal lactoferrin (Table 1).

**Table 1:** Lab Investigations; Inflammatory Markers

Investigation	Patient Value	Reference Range	Interpretation
Fecal Calprotectin	>500 µg/g	<50 µg/g (normal)	Markedly elevated
Fecal Lactoferrin	>7 µg/mL	≤4.4 µg/g (normal)	Elevated

The patient received broad-spectrum intravenous antibiotics for acute diverticulitis and suspected infectious colitis/proctitis; oseltamivir was also given for a confirmed influenza A infection. Hydrocortisone enemas (100 mg twice daily) were initiated by gastroenterology for rectal inflammation. Colorectal and plastic surgery services were also consulted, and both recommended conservative management, deferring operative intervention. On hospital day four, the patient developed acute urinary retention; the urology service inserted a Foley catheter and identified a chronic 2 cm ventral enterocutaneous fistula dating back to the 1990s, which the patient had previously declined to have repaired. His HS lesions persisted, and plastic surgery and dermatology services provided wound care and lifestyle counseling. By hospital day seven, the bleeding and inflammatory markers had improved. He was discharged on day 10 with a plan for an outpatient colonoscopy to further evaluate the sigmoid colon mass and chronic proctitis. He was also advised to follow up with the gastroenterology clinic to begin anti-TNF therapy, pending confirmation of an underlying IBD, given the efficacy of TNF-α inhibitors in managing both HS and IBD.

## Discussion

In a patient with longstanding HS, the simultaneous presence of active chronic proctitis and multiple perianal fistulous tracts presents a significant diagnostic and therapeutic challenge. The bleeding per rectum in this case can be related to either perianal HS, IBD, infectious proctitis/colitis, diverticular bleeding, colorectal cancer relapse, and/or hemorrhoidal disease. Although hemorrhoids and anal fissures are frequent benign causes of rectal bleeding, more serious conditions must always be considered, especially in high-risk patients. Infectious proctitis may present with rectal pain, discharge, and bleeding, and can be caused by Neisseria gonorrhoeae, Chlamydia trachomatis, Treponema pallidum, herpes simplex virus, or Clostridium difficile [16]. Colorectal cancer relapse, particularly in patients with prior malignancy, should be excluded with appropriate imaging and surveillance colonoscopy. Perianal HS can itself lead to bleeding through friable sinus tracts, granulation tissue, fistulizing extension, recurrent inflammation, scarring, and distortion. Importantly, HS is strongly linked to IBD, with patients showing 2.1-fold increased odds of CD and 1.5-fold increased odds of UC compared with controls [14]. Shared mechanisms-genetic predisposition, microbiome dysbiosis, and IL-23/Th17-driven inflammation-highlight a common inflammatory basis [15]. When patients with HS present with perianal symptoms, differentiating the condition from primary perianal CD is challenging due to overlapping symptoms and clinical features [15]. Furthermore, patients with both HS and IBD tend to require earlier and more aggressive immunomodulatory treatment with biologics such as anti-TNF agents offering dual benefit for skin and intestinal inflammation [17]. Histologic findings such as crypt abscess formation and lamina propria infiltration support a diagnosis of chronic colitis, keeping IBD high on the differential diagnosis. Diverticular disease is the most common cause of lower gastrointestinal bleeding and typically presents with painless, large-volume hematochezia. While diverticular bleeding often resolves spontaneously, persistent or recurrent episodes may require endoscopic intervention [18]. For a patient with diverticula and mucosal ulceration, differentiating between diverticulitis and an IBD flare, such as CD or UC, relies heavily on specific histologic markers observed in biopsy samples [19]. A multidisciplinary approach is essential, with gastroenterology guiding potential biologic therapy, dermatology managing HS, colorectal/plastic surgery teams addressing bleeding and structural complications, and urology involved in fistula care. Early recognition of IBD in the context of HS can facilitate prompt anti-TNF therapy, which has demonstrated benefit in both cutaneous and intestinal inflammation [17]. Conversely, attributing bleeding solely to infectious colitis/proctitis, hemorrhoids, and/or diverticulitis may delay critical immunomodulatory treatment. 

## Conclusions

The evidence to date strongly supports an association between HS and IBD. Research indicates patients with either condition have a significantly increased risk of developing the other. Clinicians should maintain a high index of suspicion for underlying IBD in patients with HS presenting with elevated inflammatory biomarkers and recurrent rectal bleeding, particularly when histopathology shows active chronic proctitis with focal crypt abscess formation and erosion. A coordinated multidisciplinary strategy is essential to address overlapping inflammatory processes in the skin and gut. 
